# The power of light: Impact on the performance of biocontrol agents under minimal nutrient conditions

**DOI:** 10.3389/fmicb.2023.1087639

**Published:** 2023-02-02

**Authors:** Maria E. Karlsson, Maria Hellström, Adam Flöhr, Karl-Johan Bergstrand, Beatrix W. Alsanius

**Affiliations:** Department of Biosystems and Technology, Swedish University of Agricultural Sciences, Alnarp, Sweden

**Keywords:** biocontrol agent, biofilm formation, biosurfactant production, light quality, phenotypic microarray, sole carbon source utilization

## Abstract

**Background:**

The spectral distribution of light (different wavelength) has recently been identified as an important factor in the dynamics and function of leaf-associated microbes. This study investigated the impact of different wavelength on three commercial biocontrol agents (BCA): *Bacillus amyloliquefaciens* (BA), *Pseudomonas chlororaphis* (PC), and *Streptomyces griseoviridis* (SG).

**Methods:**

The impact of light exposure on sole carbon source utilization, biofilm formation, and biosurfactant production by the selected BCA was studied using phenotypic microarray (PM) including 190 sole carbon sources (OmniLog®, PM panels 1 and 2). The BCA were exposed to five monochromatic light conditions (420, 460, 530, 630, and 660 nm) and darkness during incubation, at an intensity of 50 μmol m^−2^ s^−1^.

**Results:**

Light exposure together with specific carbon source increased respiration in all three BCA. Different wavelengths of light influenced sole carbon utilization for the different BCA, with BA and PC showing increased respiration when exposed to wavelengths within the blue spectrum (420 and 460 nm) while respiration of selected carbon sources by SG increased in the presence of red light (630 and 660 nm). Only one carbon source (capric acid) generated biosurfactant production in all three BCA. A combination of specific wavelength of light and sole carbon source increased biofilm formation in all three BCA. BA showed significantly higher biofilm formation when exposed to blue (460 nm) and green (530 nm) light and propagated in D-sucrose, D-fructose, and dulcitol. PC showed higher biofilm formation when exposed to blue light. Biofilm formation by SG increased when exposed to red light (630 nm) and propagated in citraconic acid.

**Conclusion:**

To increase attachment and success in BCA introduced into the phyllosphere, a suitable combination of light quality and nutrient conditions could be used.

## 1. Introduction

The encouraging results of microbial biocontrol agents (BCA) under laboratory and small-scale conditions do not always translate into consistent biocontrol efficacy in commercial settings. Rapidly declining numbers of introduced BCA is a recurring problem in seed, root, and foliar application. Various reasons may underlie this decline, such as problematic application techniques and poor adaptation of BCA to the commercial growing environment.

The phyllosphere harbors a variety of microorganisms that have a strong impact on plant fitness and support plant growth and survival, e.g., by improved nutrient provisioning and uptake, resilience to environmental stresses, or even disease defense (Vorholt, 2012). Organic nutrient availability on leaf surfaces is an important factor governing microbial colonization. Dissolved organic compounds exuded *via* the plant cuticle serve as energy sources to sustain the metabolism of associated microbes (Yeats and Rose, 2013). Ambient conditions, i.e., temperature, ultraviolet (UV) radiation, and relative humidity, affect nutrient extrusion (Leveau, 2009, 2019). Nutrients are not exuded evenly over the leaf surface, so microbial colonization of leaves is patchy. Moreover, the amount of exuded nutrients is finite and therefore introduced microbes need to compete with the leaf microbiota for these nutrients. Survival and establishment of BCA on the leaf surface is dependent on their ability to compete with the existing microbial community (Mallon et al., 2015). In order for BCA to co-exist with existing microbial species, limited niche overlap is needed (Chase and Myers, 2011; Hawkes and Connor, 2017). However, with respect to plant pathogens and BCA, niche overlap is essential for pathogen control.

Light (different wavelength) has recently been identified as an important factor for the dynamics and function of leaf-associated microbes (Alsanius et al., 2017, 2019, 2021; Gharaie et al., 2017). Recent studies have shown that non-phototrophic microbes can also respond phenotypically to differences in light quality (Wu et al., 2013; Beattie et al., 2018; Alsanius et al., 2021; Losi and Gärtner, 2021). Photosensory proteins in bacteria, such as blue light receptor proteins, could play a crucial role in sensing and responding to light (Losi et al., 2014). Respiration, growth rate, motility, and microbial lifestyle (planktonic, sessile) vary under different light quality levels, but are also affected by nutritional conditions (Gharaie et al., 2017; Alsanius et al., 2021). Beauregard et al. (2013) demonstrated that specific polysaccharides leaching from the plant serve as a cue for *Bacillus subtilis* to form biofilm on the root of *Arabidopsis thaliana*. Biosurfactant formation by BCA is a crucial mechanism to facilitate their dispersal on the leaf surface and biofilm formation is essential for their establishment (Alsanius et al., 2021). Thus, manipulation of light quality and nutritional factors might enable BCA to transition between planktonic and sessile lifestyles, and could be a key factor for optimized BCA performance on leaf surfaces.

In controlled-environment plant production, e.g., in greenhouses, artificial irradiation with mono- or polychromatic light sources with wavelength from 400 to 700 nm is used to optimize crop photosynthesis, biomass formation, and/or plant architecture (Morrow, 2008). Monochromatic blue and red light and polychromatic white light influence phyllospheric community structure (Vänninen et al., 2010; Alsanius et al., 2017). The lethal effect of UV-light on plant pathogens is well established (Newsham, 1997; Kadivar and Stapelton, 2003). However, other wavelengths within the visible light spectrum have also been demonstrated to affect the behavior of plant pathogens such as downy and powdery mildew (Reuveni and Raviv, 1997; Suthaparan et al., 2012, 2014) and grey mold (*Botrytis cinerea*; Nicot et al., 1996; Elad, 1997). Examples of light spectra-dependent performance have also been reported for non-pathogenic microorganisms, such as *Pseudomonas* sp. DR 5-09 (Gharaie et al., 2017) and *Bacillus amyloliquefaciens* (Yu and Lee, 2013; Rajalingam and Lee, 2017).

The aim of this study was to determine the effects of different wavelengths on utilization of different sole carbon sources by commercial BCA and their biosurfactant production and biofilm formation. The starting hypotheses were that: (i) different wavelength affects the substrate utilization pattern of the target strains; and (ii) different wavelength affects biofilm formation and biosurfactant production by the target strains.

## 2. Materials and methods

The study was conducted using three commercial BCA strains. *Bacillus amyloliquefaciens* DSM7 (BA) and *Pseudomonas chlororaphis* 50083 (PC) were purchased from DSMZ (Leibniz Institute, Braunschweig, Germany). *Streptomyces griseoviridis* CBS904.68 (SG) was purchased from Centraalbuureau voor Schimmelcultures, Utrecht, Netherlands.

### 2.1. Phenotypic microarray

Phenotypic microarrays (PM) were performed following procedures described by Gharaie et al. (2017) and Alsanius et al. (2021). In brief, the microarrays were performed at a density of six replicates per strain and treatment on two sole carbon source panels (PM01, PM02A) according to the Biolog standard protocols, using 190 different sole carbon sources. Bacteria were propagated overnight from cryoculture at 25°C on tryptic soy agar (TSA; DIFCO 236950, United States). Colony swabs were transferred to IF-0a GN medium (Biolog Inc., Hayward, CA, United States) and turbidity of the bacterial suspension was adjusted turbidimetrically (Biolog Inc., United States, catalog no. 3587) to 81% transmittance. Redox dye (dye mix A (catalog no. 74221) for PC; dye mix G (catalog no. 74227) for BA and SG; Biolog Inc., Haywood, United States) was then added. A 100 μl aliquot of the suspension was pipetted into each plate well and the plates were sealed with Greiner ViewSeal (Greiner Bio-one, 676070; Sigma Aldrich, Z617571-100EA, St. Louis, MO, United States; Gharaie et al., 2017). The panels were exposed to five monochromatic light-emitting diode (LED) light regimes (blue: 420 and 460 nm, green: 530 nm, red: 630 and 660 nm), while control panels were incubated in darkness for 96 h. Panels incubated in darkness were kept in the OmniLog incubator (OmniLog, catalog number 93182, Biolog Inc., United States) at 20°C during the entire incubation period. Panels exposed to the various light conditions were placed in lined cabinets (500 × 500 × 1,000 mm) and incubated at 20°C. Each cabinet was equipped with a LED lamp (Heliospectra Dyna, Heliospectra AB, Gothenburg, Sweden). Light intensity was adjusted to 50 μmol m^−2^ s^−1^.

Sole carbon source utilization in each well of the PM panels was measured as color change in the added redox dye, using a computer-controlled camera system. Under dark conditions, color change was recorded automatically every 15 min, while under light exposure color change of the PM panels was recorded according to previously established growth curves. Readings were set to occur 0, 6, 10, 24, 30, 48, 54, 72, and 96 h post inoculation (hpi). Output values were expressed in OmniLog units.

### 2.2. Biosurfactant production

Biosurfactant formation was monitored using a drop collapse test. Aliquots of 20 μl from each well of the carbon source panels (PM01 and PM02A) were transferred to glass plates covered with parafilm and a template of the 96-well plate. After 2 min, each drop was scored from 0 to 2 (0 = convex, 1 = moderately convex and 2 = flattened drop; Gharaie et al., 2017).

### 2.3. Biofilm formation

Assessment of biofilm formation followed the procedure described by Alsanius et al. (2021). In brief, the microbial suspension was removed from the PM panels, the plates were washed, and 100 μl of 0.5% crystal violet solution (1% Crystal violet solution, V5265-500ML, Sigma-Aldrich) were added to each well, after which the plates were incubated for 15 min. The crystal violet solution was then removed and the plates were repeatedly washed and left to dry overnight. Finally, 100 μl of 95% ethanol were added to each well and the plates were left for 60 min before spectrophotometric determination of extinction at 550 nm (Expert 96TM spectrophotometer, AsysHiTech, Eugendorf, Austria).

### 2.4. Analysis and statistical calculations

The recorded carbon utilization data were exported as csv-files using OmniLog PM kinetic analysis software and then analyzed in R-studio using the *opm* package (Vaas et al., 2013; Göker et al., 2016; R Core Team, 2021), based on curve parameter maximum curve height (A) and area under curve (AUC). Analysis of variance (ANOVA) was used to analysis the biofilm formation data, followed by Tukey test, both performed in R-studio.

## 3. Results

### 3.1. Sole carbon utilization

**Figure 1:**
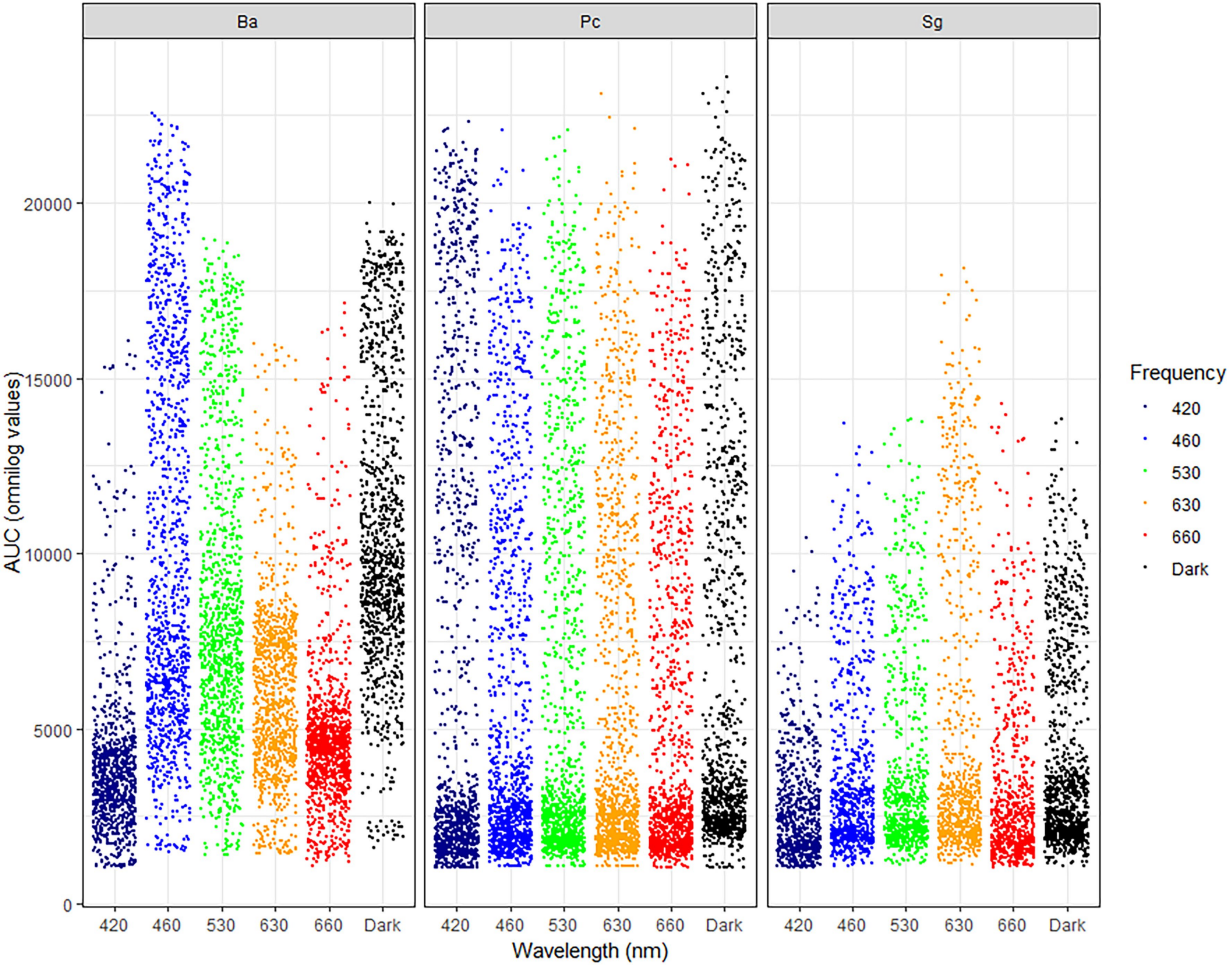
Jitter plot showing the area under the curve (AUC) for utilization of 190 sole carbon sources under different wavelengths (420, 460, 530, 630, and 660 nm) and in dark conditions by *Bacillus amyloliquefaciens* (BA), *Pseudomonas chlororaphis* (PC), and *Streptomyces griseoviridis* (SG). Each dot represents maximum Omnilog value of each single carbon source.

**Figure 2:**
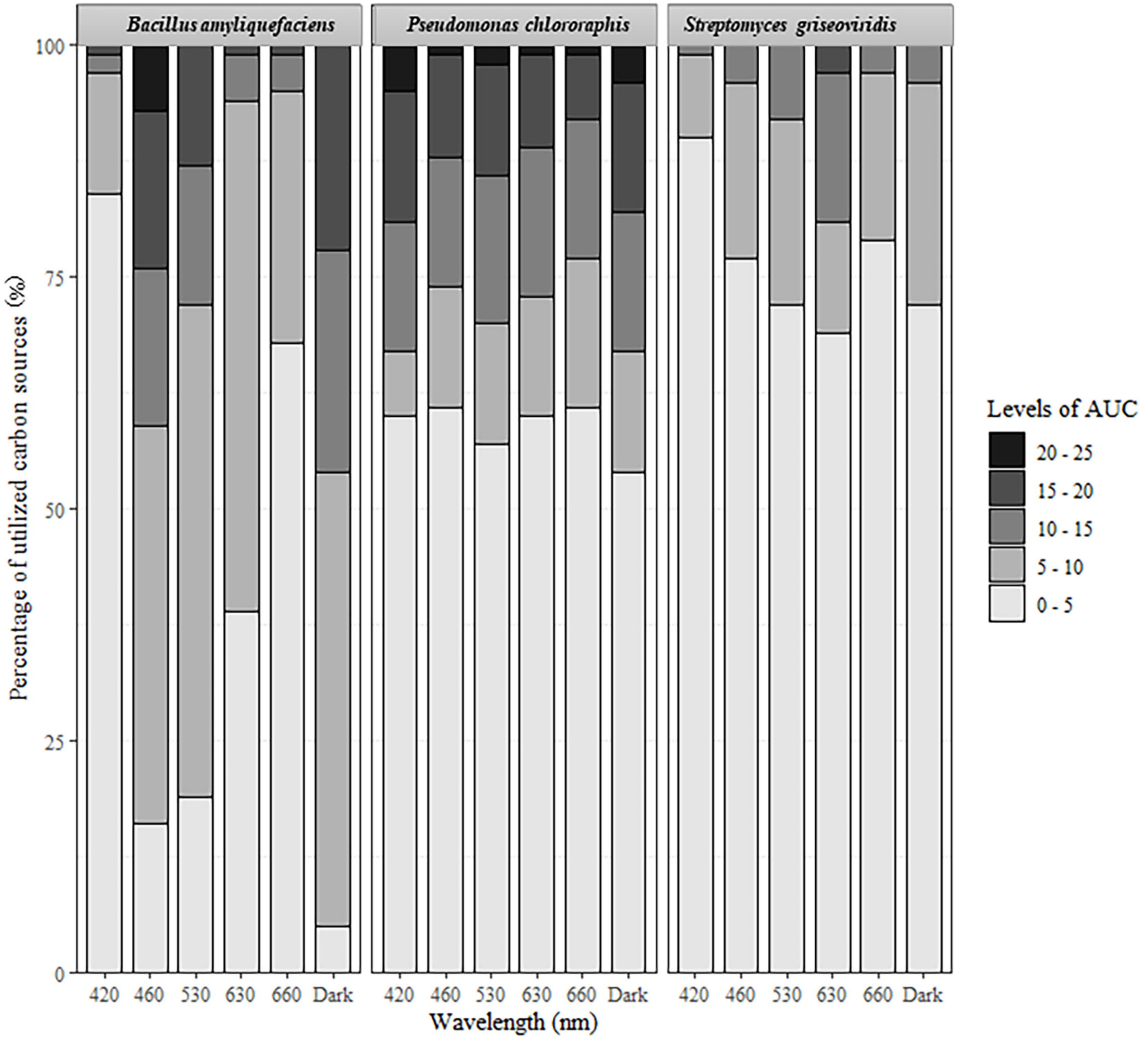
Percentage of carbon sources utilized under different light regimes. Area under the curve (AUC) values ranged from 0 to 25,000.

**Figure 3:**
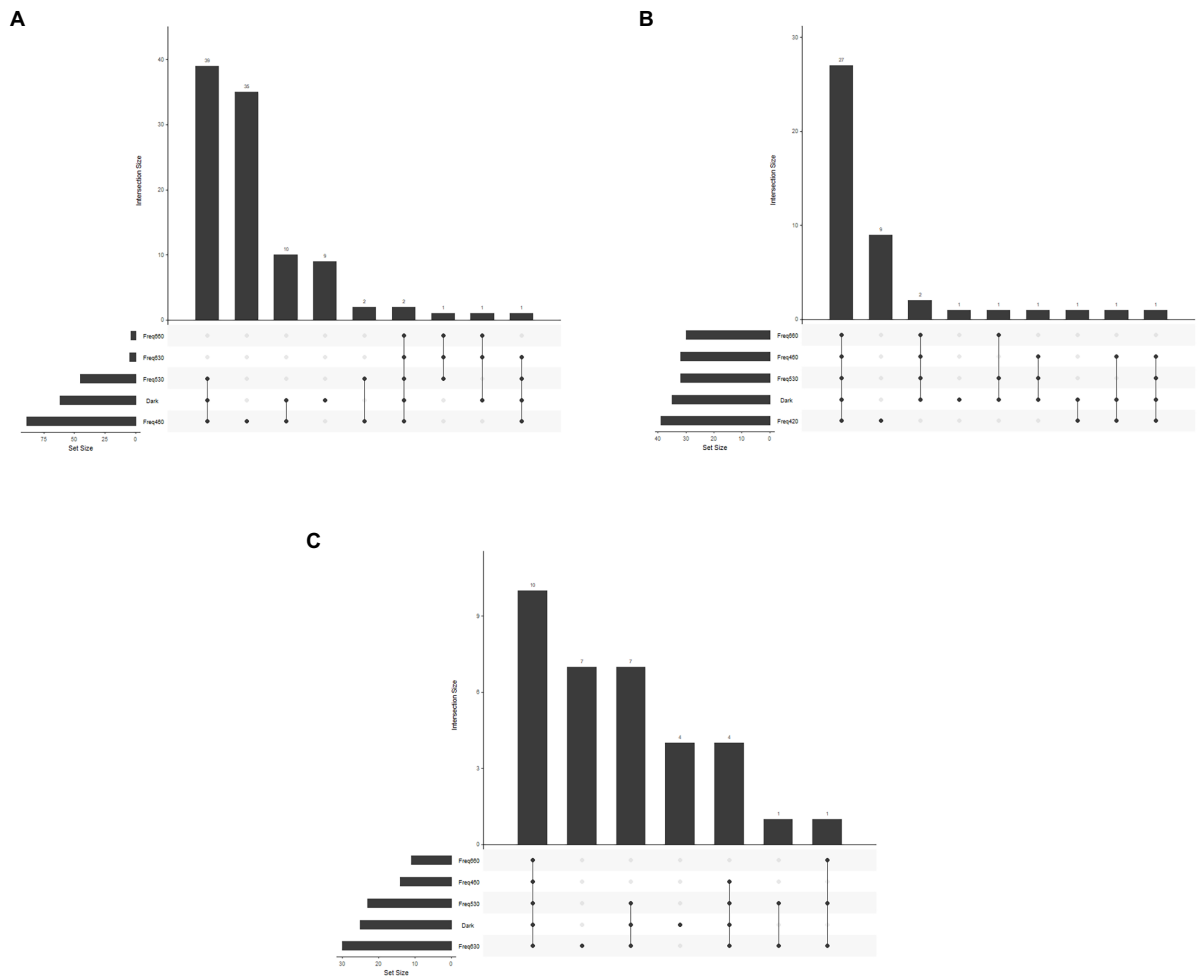
Upset plot of number of carbon sources utilized under different wavelengths and number of carbon sources utilized under the same wavelength. **(A)** *Bacillus amyloliquefaciens*, **(B)** *Pseudomonas chlororaphis*, and **(C)** *Streptomyces griseoviridis*.

For all three test-organisms, sole carbon utilization changed when the bacteria were exposed to different light regimes. No directionality was identified, since the impact of light on the respiration was increased on some carbon sources and decreased on other. For BA, the number of utilized carbon sources and intensity of utilization were generally highest on exposure to 460 and 530 nm (blue and green spectrum) and corresponded to the utilization pattern under dark conditions (Figure 1). Blue light exposure increased respiration by BA of 7% of the carbon sources to a level above AUC 20000 (Figure 2), which was not observed under dark conditions. Based on the KEGG database, the carbon sources for BA affected by light treatment were those responsible for amino sugar and nucleotide metabolism, and biosynthesis of secondary metabolites and antibiotics, and involved in the phosphotransferase system. BA exhibited generalist behavior with respect to almost all carbon sources tested when exposed to blue light (460 nm). In total, 39 carbon sources were utilized by BA under the 460 nm and 530 nm treatments and in the dark incubation. Two carbon sources (Tween 20 and Tween 40) were utilized under all wavelengths except 420 nm (Figure 3A; Supplementary Figures S1, S2).

Sole carbon utilization by PC was less sensitive to the different light regimes tested, as multiple carbon sources were consumed under all light conditions. However, there was a trend for increased utilization in the near-blue spectrum of 420 nm (Figure 1), which increased utilization of 5% of the carbon sources (Figure 2). PC showed increased respiration for the sole carbon sources involved in biochemical processes mentioned above for BA, but also for carbon sources responsible for inositol phosphate metabolism and bacterial chemotaxi. In total, 27 carbon sources (L-glutamine, mucic acid, L-aspargine, inosine, D-mannitol, fumaric acid, D-saccharic acid, D-gluconic acid, Ala-Gly, D-serine, D,L-malic acid, L-alanine, citric acid, Gly-Glu, succrose, L-serine, D-malic acid, L-malic acid, D-trehalose, myo-inositol, L-aspartic acid, Gly-Pro, L-proline, D-mannose, *N*-acetyl-D-glucosamine, pyruvic acid, D-aspartic acid) were utilized by PC under all light treatments (Figure 3B; Supplementary Figures S1, S2).

In general, sole carbon source utilization by SG was very low for almost all carbon sources tested (Supplementary Figures S1, S2) under light and dark exposure. From this low utilization rate, an increase in utilization was found under exposure to the red spectrum (630–660 nm; Figure 1). Respiration by SG rose to an AUC level of 15,000 only on 3% of the carbon sources tested (inosine, α-D-glucose-1-phosphate, D-glucose-6-phosphate, D-galcturonic acid, D-glucoronic acid, D-fructose-6-phosphate; Figure 2). These carbon sources are involved in various metabolic pathways, such as starch and sugar metabolism, biosynthesis of secondary metabolites, ABC transport, and D-amino acid metabolism. Ten carbon sources were utilized under all light spectra and seven carbon sources were solely utilized under the red spectrum of 630 nm (Figure 3C; Supplementary Figures S1, S2).

### 3.2. Biosurfactant production

Only one of the 190 carbon sources tested (capric acid) generated drop collapse in all three BCA, regardless of high or low respiration level (S2). Hence, different wavelengths had an effect on surface activity. Upon dark incubation, complete drop collapse was noted for BA and SG, whereas PC showed moderately convex droplets. No drop collapse was noted for the incubated capric acid suspension when BA was exposed to 530 nm or when PC was exposed to 630/660 nm. Moderate drop collapse was found after incubation of SG in capric acid solution exposed to red light (620 nm), but no drop collapse was seen on exposure to blue light (400, 420 nm; Table 1).

**Table 1:** Drop collapse of sole capric acid suspensions incubated with *Bacillus amyloliquefaciens, Pseudomonas chlororaphis*, or *Streptomyces griseoviridis* for 96 h under exposure to different light regimes (monochromatic LED at 400, 430, 460, 530, 620, and 660 nm; dark conditions).

Wave length	*Bacillus amyloliquefaciens*	*Pseudomonas chlororaphis*	*Streptomyces griseoviridis*
Dark	2	1	2
400	2	2	1
430	2	2	1
460	2	2	2
530	0	2	0
620	2	0	0
660	2	0	2

0 = convex, 1 = moderately convex, and 2 = flattened drop.

### 3.3. Biofilm formation

**Figure 4:**
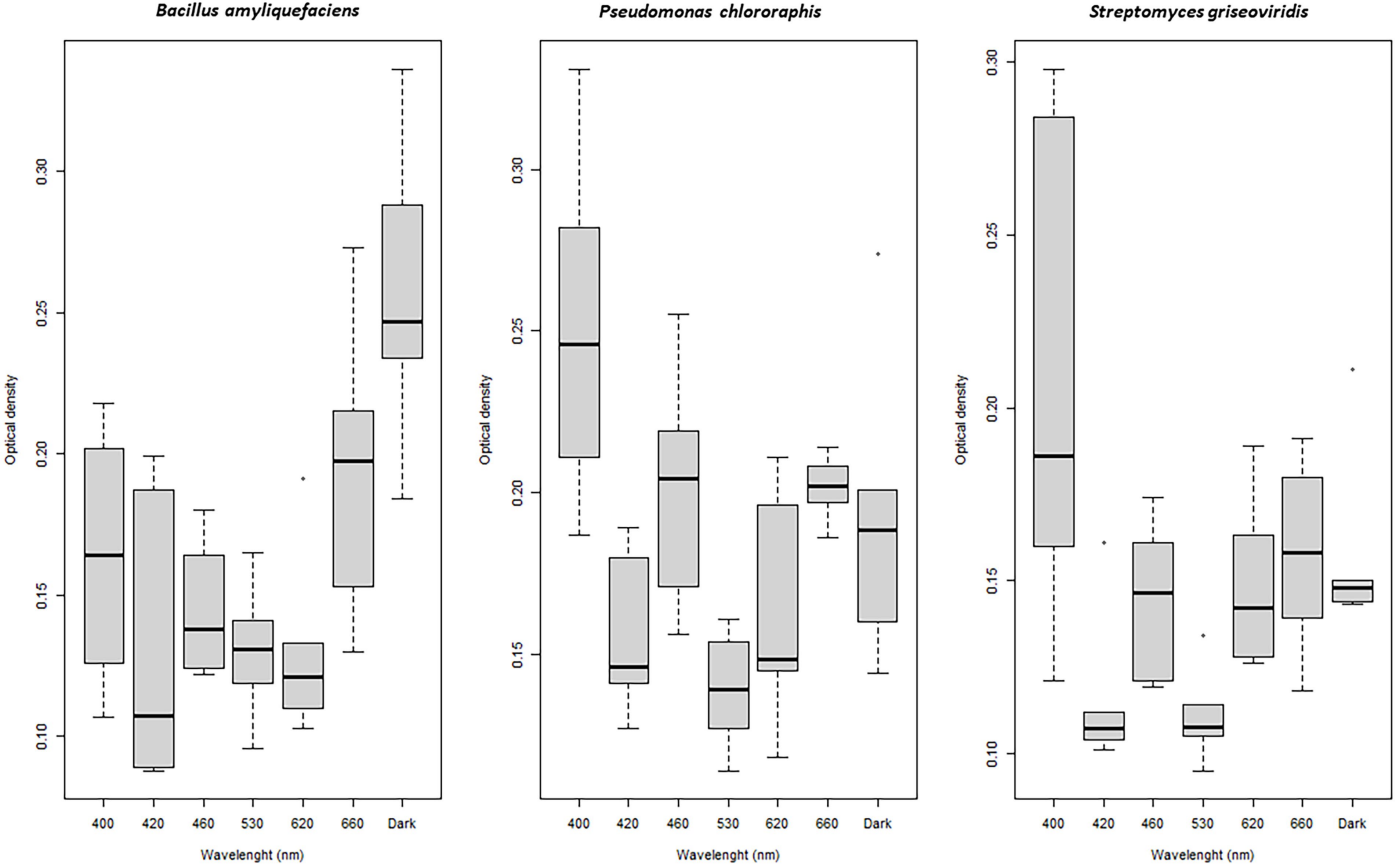
Boxplot of biofilm formation by *Bacillus amyloliquefaciens*, *Pseudomonas chlororaphis*, and *Streptomyces griseoviridis* after 96 h of exposure to different wavelenghts (biofilm measured as optical density at 550 nm).

Bacterial biofilm formation was affected by light quality. Biofilm formation by BA was enhanced when this species was incubated under dark and red (660 nm) light conditions (*p* < 0.01; Figure 4). A combination of light quality and specific carbon source increased biofilm formation in some cases, e.g., BA showed significantly higher biofilm formation when exposed to blue (460 nm) and green (530 nm) light and propagated in D-sucrose, D-fructose, and dulcitol, together with a very high respiration level (Table 2).

**Table 2:** Directionality of biofilm formation by *Bacillus amyloliquefaciens*, *Pseudomonas chlororaphis*, and *Streptomyces griseoviridis* in the presence of selected sole carbon sources under exposure to different light regimes (light intensity: 50 μmol s^−1^ m^−1^: monochromatic LED: 400, 420, 460, 530, 620, and 660 nm; darkness).

Wave length	*Bacillus amyloliquefaciens*		*Pseudomonas chlororaphis*		*Streptomyces griseovirides*	
400			Glycolic acid	↓		
420					α-Methyl-D-Mannoside	↓
460	D-Sucrose	↑	α-D-Glucose	↑		
	Dulcitol	↑	m-Tartaric acid	↑		
			Uridine	↑		
			
530	D-Sucrose	↑	D-Malic acid	↑	L-Alanine	↓
	D-fructose	↑	δ-Amino valeric acid	↓		
			Citramalic acid	↓		
			Capric acid	↓		
			
620			D-Ribose	↑	Citraconic Acid	↑
			Sorbic Acid	↓	L-Rhamnose	↓
			δ-Amino valeric acid	↓		
			Citramalic acid	↓		
			
660	Dulcitol	↑	D-Ribose	↑		
			δ-Amino valeric acid	↓		
			Sorbic acid	↓		
			Citramalic acid	↓		
Dark			D-Ribose	↑		
			Sorbic acid	↓		

Arrows indicate the directionality of carbon source utilization after 96 h of incubation (OmniLog value; ↑ = high respiration, ↓ = low respiration).

PC showed significantly (*p* < 0.001) higher biofilm formation when exposed to blue (400 nm) light compared with dark conditions (Figure 4). PC had higher respiration levels when grown on the carbon sources α-D-glucose, m-tartaric acid, uridine, D-malic acid, and D-ribose, where light quality seemed to be of minor importance (Table 2).

Biofilm formation by SG increased significantly under blue (400 nm) light (*p* = 0.04) compared with dark conditions (Figure 4). SG showed higher respiration levels and high biofilm formation when propagated on citraconic acid as the carbon source and exposed to red light (630 nm). Biofilm formation was still found when SG was exposed to 530 nm with L-alanine, 630 nm with L-rhamnose, and 420 nm with α-methyl-D-mannoside as the carbon source, despite low respiration level (Table 2).

## 4. Discussion

The results obtained in this study confirm that metabolism in non-phototrophic bacteria is altered by the prevailing light environment and that directionality of change is absent, as previously suggested by Gharaie et al. (2017) and Alsanius et al. (2021). From an ecological perspective, application of BCA to a crop can be seen as a microbial invasion, which is a widespread phenomenon in nature. This phenomenon follows a certain process, starting with (I) introduction, when the invader comes into a new environment, followed by (II) establishment of the invader and maintenance of a viable invader population. After establishment, the invader has to (III) disperse in the new environment and if successful it can displace or re-shape the resident microbial community (Mallon et al., 2015). Another important aspect is competition for resources. When using BCA to control plant pathogens, a certain niche overlap is desirable. In theory, niche overlap means that species have similar factors that regulate their population growth, such as nutrients and response to different stressors in the environment (Pastore et al., 2021). In the present study, we selected three commercially available BCA with known efficacy against powdery mildew and grey mold and investigated whether habitat manipulation could enhance establishment of the selected species. Based on sole carbon source utilization, the results demonstrated that the phenotypic plasticity of the selected BCA varies under minimal nutrient conditions and that directionality in phenotypic response is dependent on (i) the bacterial strain, (ii) the light spectrum, and (iii) the individual carbon source (Supplementary Figures S1, S2). In general, based on sole carbon source utilization, PC and BA showed high variable plasticity in response to the light spectrum and were particularly enhanced by blue light, while SG showed low plasticity, with low respiration rates and low sensitivity to the different spectra, although red light increased sole carbon source utilization by SG to some extent.

The findings for PC support previous findings by Gharaie et al. (2017) and Alsanius et al. (2021). However, it is worth noting that different species within the genus *Pseudomonas* differ in their carbon source utilization rate, with respect to intensity and maximum utilization. Thus, the impact of blue light needs to be determined separately for different pseudomonad strains before the insights in this study are used for secondary purposes, such as improved metabolite formation. This is also true for species within *Bacillus*. In contrast to *Bacillus thuringiensis* (Alsanius et al., 2021), in the present study BA displayed light sensitivity as assessed by respiration under minimal nutrient conditions. It is also worth noting that phenotypic plasticity is a rapid adaptation response to a threat in the environment, enabling growth and propagation (Chevin and Hoffmann, 2017). In this study, different wavelength and nutrient conditions changed and thus the bacteria needed to change their utilization pattern and adapt to the new challenging environment if they were to grow and survive.

Biosurfactant production and biofilm formation are essential mechanisms for dispersal and establishment of BCA on a given surface. Only one (capric acid) of the 190 sole carbon sources tested in this study induced biosurfactant production in all three BCA, but under different lighting conditions. However, other studies have reported effects of various organic compounds, such as carbohydrates and amino acids, in enhancing biosurfactant production (Guerra-Santos et al., 1986; Alsanius et al., 2021). In the study by Alsanius et al. (2021), no drop collapse was observed at lower respiration levels, whereas in our study drop collapse was observed at very low respiration rates for SG.

Different light spectra influence physiological responses and metabolic pathways in microorganisms, such as swarming motility, biofilm formation, virulence, and antibiotic production (Kraiselburd et al., 2012; Yu and Lee, 2013; Müller et al., 2017). From an agriculture and horticulture point of view, biofilm formation and BCA dispersal are crucial for the control of microbial pathogens and for the overall utility of BCA. Therefore, it is important to determine the role of light in bacterial behavior. Non-phototrophic bacteria may be equipped with photosensory proteins, which are involved in controlling the transition between a planktonic lifestyle and a sessile multicellular lifestyle in biofilm (van der Horst et al., 2007; Purcell and Crosson, 2008). These blue-light receptors are linked to two protein domains (GGDEF and EAL) that have been shown to control this transition through cyclic di-GMP, a second messenger that stimulates the biosynthesis of adhesins and poly-saccharide matrix substances important for biofilm formation (Jenal and Malone, 2006). Organic compounds such as acyl-homoserine lactones (AHL) and phenazines have been shown to be strongly linked to biofilm formation (Rieusset et al., 2020).

**Figure 5:**
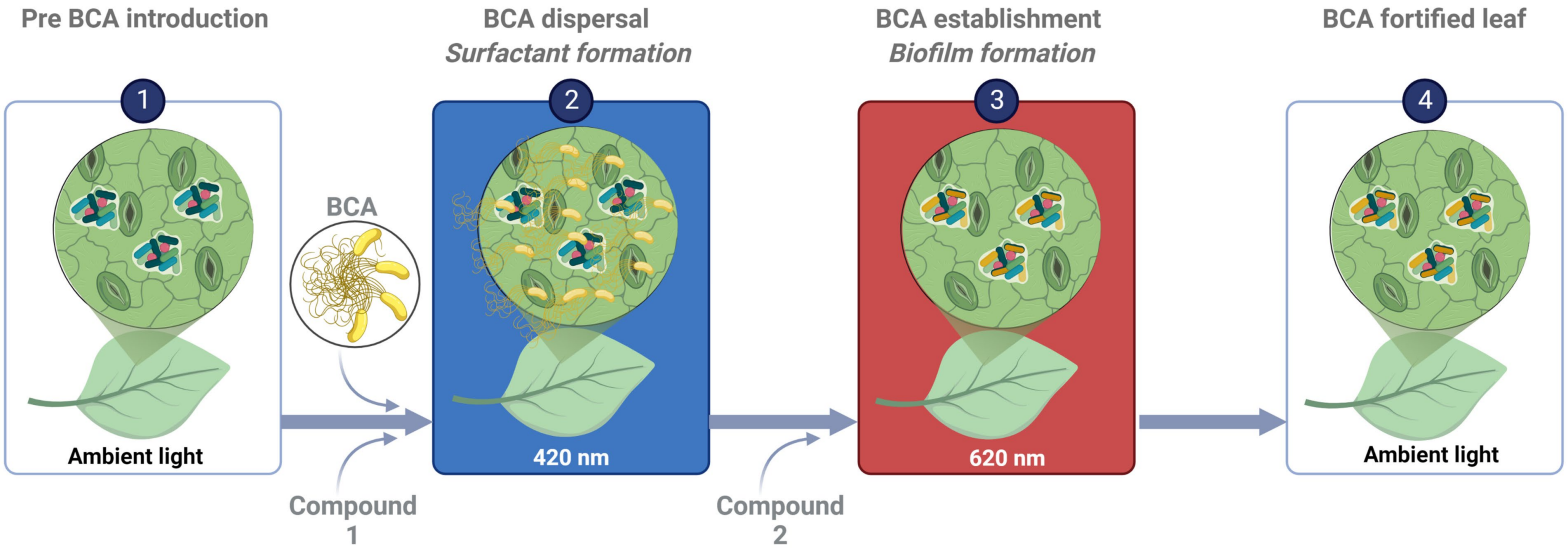
Schematic diagram illustrating putative areas of application for biocontrol agents (BCA). *(Illustration: B. Alsanius; “Created with* BioRender.com*.” agreement number HJ24LJ7YZ0).*

The phyllosphere is often described as a challenging environment for microbiota, especially with respect to the availability of organic nutrients. We therefore applied a minimal nutrient approach to mimic such conditions. To translate the findings to greenhouse settings and improve the establishment and efficacy of BCA, challenge experiments need to be conducted in planta under greenhouse conditions. One approach could be to apply BCA together with a specific carbon source and light quality that trigger BCA dispersal and surfactant production, followed by another compound and light quality that enhance establishment and biofilm formation (Figure 5).

In conclusion, our results showed that the choice of wavelength affects the sole carbon source utilization pattern of all three target strains and metabolic responses to a particular wavelength was species-specific. Biosurfactant production and biofilm formation are important mechanisms for BCA to be successful. To use different wavelengths of light may enhance establishment of BCA on the leaf surface. The findings need to be validated under greenhouse conditions.

## Data availability statement

The raw data supporting the conclusions of this article will be made available by the authors, without undue reservation.

## Author contributions

MK and BA contributed to the conception of the study and the design, and wrote the original draft. AF contributed statistical support and helped in managing the opm package. MH and K-JB critically reviewed the manuscript. MK and MH performed the experiments. MK conducted data analysis. BA acquired funding. All authors contributed to the article and approved the submitted version.

## Funding

This research was funded by Stiftelsen Lantbruksforskning and the Swedish Research Council for Environment, Agricultural Sciences and Spatial Planning, both Stockholm, Sweden, under grant number R-18-25-006 (“Optimized integrated control in greenhouse systems sees the light”; PI: BA).

## Conflict of interest

The authors declare no conflict of interest. This research was conducted in the absence of any commercial or financial relationships that could be construed as a potential conflict of interest.

## Publisher’s note

All claims expressed in this article are solely those of the authors and do not necessarily represent those of their affiliated organizations, or those of the publisher, the editors and the reviewers. Any product that may be evaluated in this article, or claim that may be made by its manufacturer, is not guaranteed or endorsed by the publisher.

## Supplementary material

The Supplementary material for this article can be found online at: https://www.frontiersin.org/articles/10.3389/fmicb.2023.1087639/full#supplementary-material

SUPPLEMENTARY FIGURE S1 Overview of the utilization pattern of *B. amyloliquefaciens, P. chlororaphis* and *S. griseoviridis* on 96 carbon sources (PM01). The heatmap shows maximum curve hight values when exposed to LED with the wavelenght 420, 460, 530, 630, 660 nm and dark. The legend explains the color code from blue to green to yellow indicate low, moderate and high substrate utilization levels.

SUPPLEMENTARY FIGURE S2 Overview of the utilization pattern of *B. amyloliquefaciens, P. chlororaphis* and *S. griseoviridis* on 96 carbon sources (PM02). The heatmap shows maximum curve hight values when exposed to LED with the wavelenght 420, 460, 530, 630, 660 nm and dark. The legend explains the color code from blue to green to yellow indicate low, moderate and high substrate utilization.
