# Supplementary figures and images for: The power of light: Impact on the performance of biocontrol agents under minimal nutrient conditions
Source: Front Microbiol. 2023 Feb 2;14:1087639. doi: 10.3389/fmicb.2023.1087639 (PMC9932321; doi:10.3389/fmicb.2023.1087639)

## and Histogram

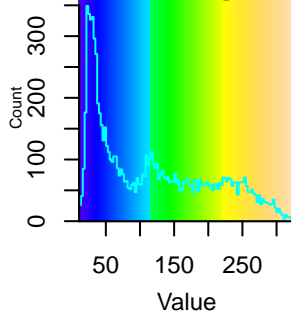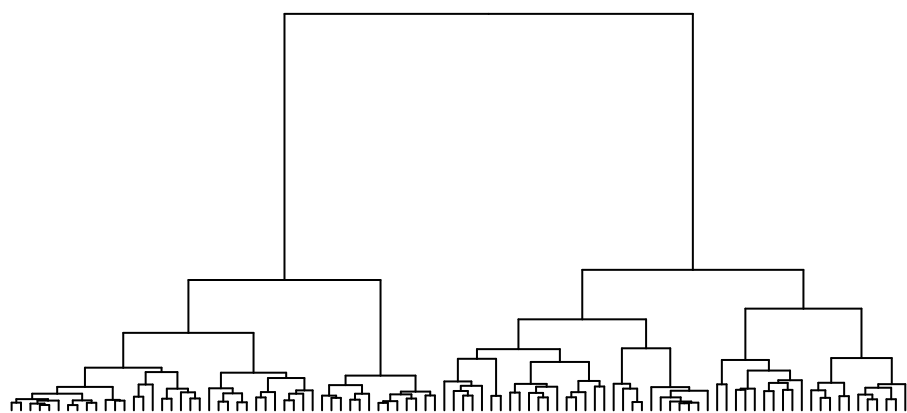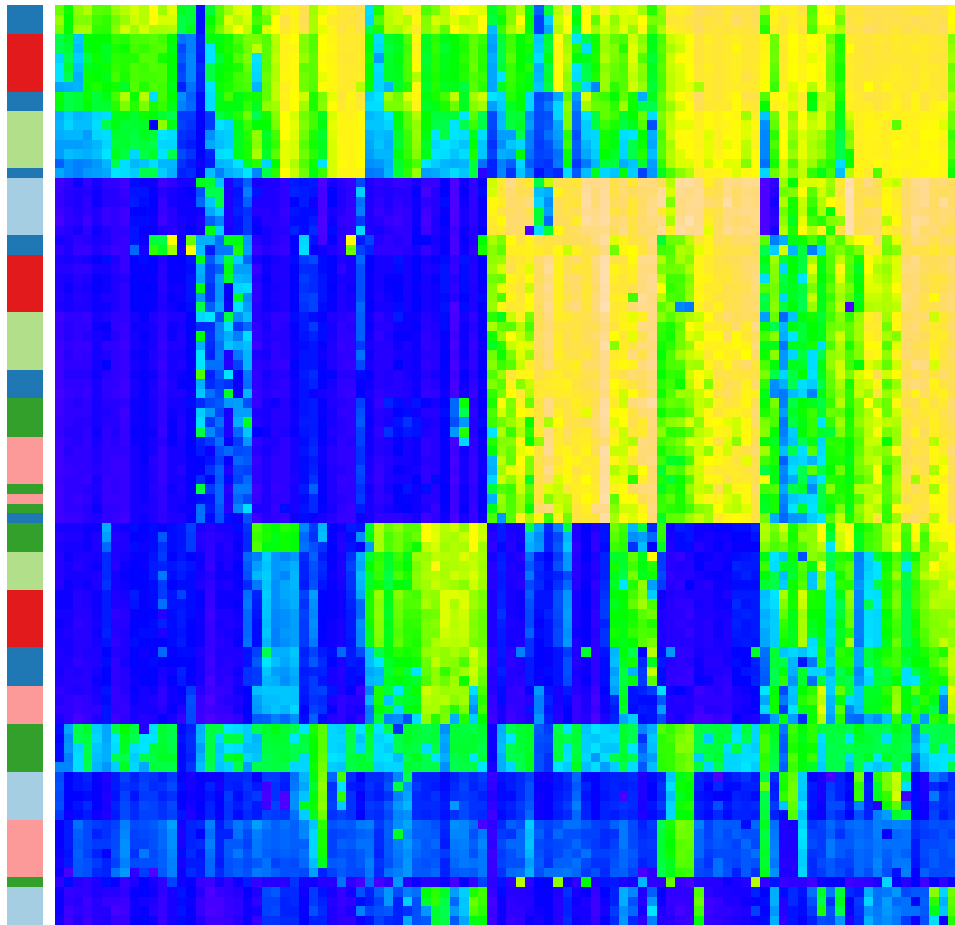[illegible]

Supplement: SUPPLEMENTARY FIGURE S1 — Overview of the utilization pattern of B. amyloliquefaciens, P. chlororaphis and S. griseoviridis on 96 carbon sources (PM01). The heatmap shows maximum curve hight values when exposed to LED with the wavelenght 420, 460, 530, 630, 660 nm and dark. The legend explains the color code from blue to green to yellow indicate low, moderate and high substrate utilization levels. [file Data_Sheet_1.PDF]

## and Histogram

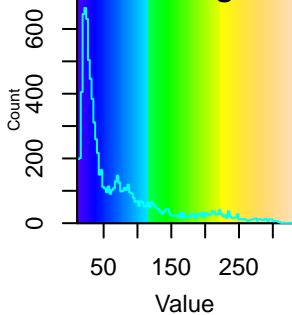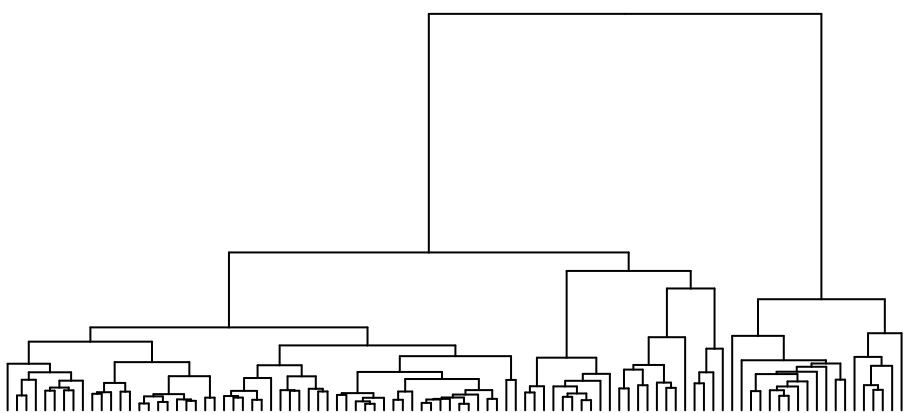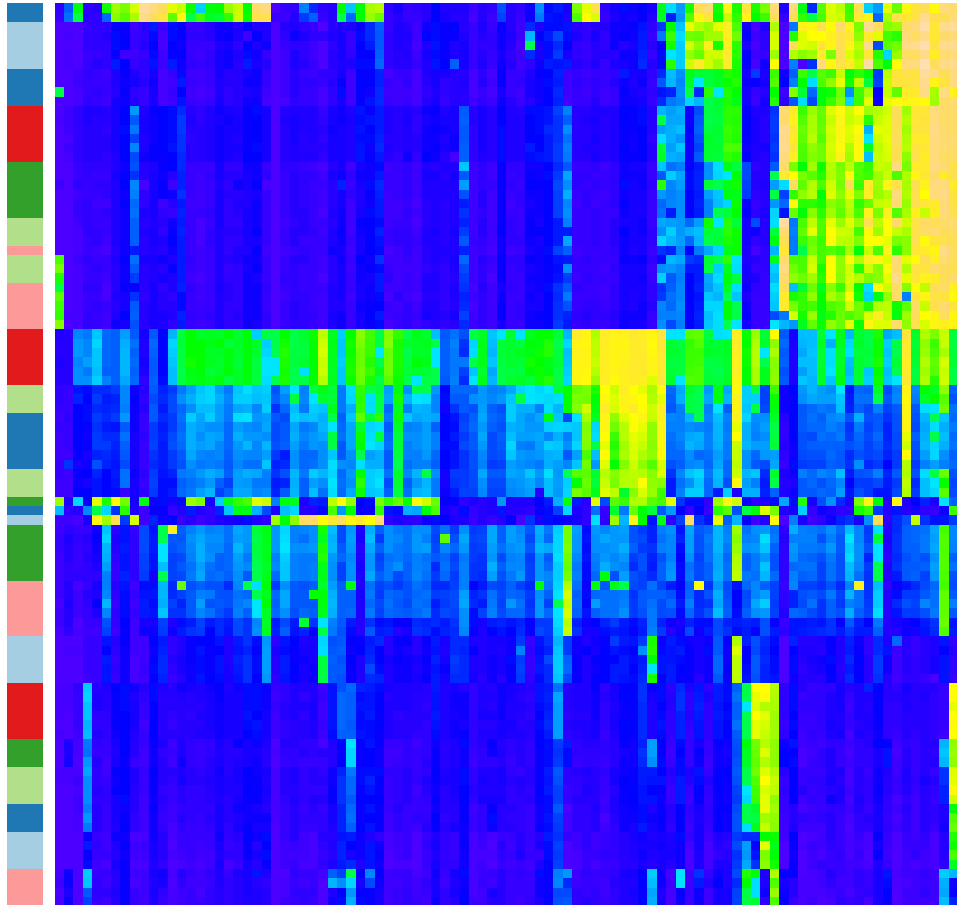[illegible]

Supplement: SUPPLEMENTARY FIGURE S2 — Overview of the utilization pattern of B. amyloliquefaciens, P. chlororaphis and S. griseoviridis on 96 carbon sources (PM02). The heatmap shows maximum curve hight values when exposed to LED with the wavelenght 420, 460, 530, 630, 660 nm and dark. The legend explains the color code from blue to green to yellow indicate low, moderate and high substrate utilization. [file Data_Sheet_2.PDF]
